# [Retracted] MicroRNA‑599 targets high‑mobility group AT‑hook 2 to inhibit cell proliferation and invasion in clear cell renal carcinoma

**DOI:** 10.3892/mmr.2023.13041

**Published:** 2023-06-26

**Authors:** Hailing Zhao, Huizhen Zhao, Xiaolin Xia, Xiujuan Liu

Mol Med Rep 17: 7451–7459, 2018; DOI: 10.3892/mmr.2018.8755

Following the publication of the above paper, it was drawn to the Editors’ attention by a concerned reader that cell migration and invasion assay data shown in Fig. 5C were strikingly similar to data appearing in different form in other articles by different authors, which have been retracted. Owing to the fact that the contentious data in the above article were already under consideration for publication, or had already been published, elsewhere when it was submitted to *Molecular Medicine Reports*, the Editor has decided that this paper should be retracted from the Journal. The authors were asked for an explanation to account for these concerns, but the Editorial Office did not receive any reply. The Editor apologizes to the readership for any inconvenience caused.

